# Neonatal Gut Microbiota in Puppies and Kittens: From Maternal Transmission to Immune Development

**DOI:** 10.3390/ani16091307

**Published:** 2026-04-24

**Authors:** Raquel Rodríguez-Trujillo, Miguel Batista-Arteaga, Kseniia Isupova, Sara Alonso-Santana, Alberto Acosta-Urbano, Xiomara Lucas-Arjona, Soraya Déniz-Suárez

**Affiliations:** 1 Unit of Reproduction, Universitary Institute of Biomedical Research and Health, University of Las Palmas de Gran Canaria, Transmontaña s/n 35413, 35413 Arucas, Las Palmas, Spain; raquel.rodriguez@ulpgc.es (R.R.-T.); ksfedorchak23@gmail.com (K.I.); sara.alonso@ulpgc.es (S.A.-S.); 2 Veterinary Teaching Hospital, Department of Animal Medicine and Surgery, University of Murcia, Campus de Espinardo, 30100 Murcia, Spain; albertoacostaurbano@gmail.com (A.A.-U.); xiolucas@um.es (X.L.-A.); 3 Veterinary Infectious Diseases and Ichthyopathology, Veterinary School, University of Las Palmas de Gran Canaria, 35413 Arucas, Las Palmas, Spain; soraya.deniz@ulpgc.es

**Keywords:** neonatal microbiota, development, health, dysbiosis, neonatology, newborn

## Abstract

Neonatal puppies and kittens face a critical period after birth, during which their health depends heavily on the microorganisms they acquire from their mothers and environment. These microorganisms, known as the gut microbiota, help newborns develop their immune systems, digest nutrients, and protect against disease. This review explores how these microorganisms are transferred from the mother to her offspring before, during, and after birth, including the process of delivery, nursing, and maternal care. It also examines how factors such as birth type, hygiene, feeding, and maternal health can influence the development of these microbial communities. When this process is disrupted, it may lead to health problems such as infections, diarrhea, and immune disorders. Understanding how and when these microbes are passed to newborns, and how to support this process, is essential to improving survival rates and long-term health in puppies and kittens.

## Simple Summary

Neonatal puppies and kittens face a critical period after birth in which gut microbial colonization, maternal immune support, and environmental management strongly influence survival and long-term health. This review summarizes current evidence on how the neonatal gut microbiota is acquired and shaped through maternal transmission before, during, and after parturition (including delivery mode, colostrum and milk intake, maternal contact/licking, and hygiene conditions). It also discusses how disruptions to normal colonization—such as cesarean delivery, inadequate colostrum intake, maternal illness or stress, and suboptimal environmental conditions—may contribute to dysbiosis and increase the risk of neonatal disorders, including fading puppy/kitten syndrome, sepsis, diarrhea/gastroenteritis, toxic milk syndrome, and immune dysregulation. Finally, the review highlights major knowledge gaps, especially in pre-weaning kittens, and outlines priorities for standardized, longitudinal studies and targeted preventive interventions (e.g., maternal nutritional modulation and pre/probiotic strategies).

## 1. Introduction

Neonatology in small companion animals has witnessed considerable advancements in recent years, primarily driven by ongoing efforts to reduce neonatal mortality, which remains a significant concern in veterinary practice [1]. In this review, “small companion animals” refers specifically to domestic dogs and cats (puppies and kittens). Early identification and management of risk factors are essential to improving survival outcomes in neonatal puppies and kittens [2]. Among these, cesarean section (CS) is frequently employed as a preventive measure, especially in high-risk pregnancies, to enhance neonatal viability [3,4]. However, data on gut microbiota development remain limited in both puppies and kittens, and are particularly scarce in pre-weaning kittens. Most available studies focus on the weaning transition rather than the immediate neonatal period, highlighting a significant knowledge gap in feline neonatology [5].

The physiological and endocrine mechanisms underlying parturition play a pivotal role in neonatal outcomes. In the bitch, parturition is initiated by a prepartum decline in maternal serum progesterone, modulated by fetal and maternal endocrine cues, including increased fetal cortisol and maternal prostaglandin release [6]. These hormonal changes not only induce myometrial contractions but also contribute to placental separation and fetal expulsion. When timed from ovulation, gestation length in the bitch is relatively consistent, ranging from 64 to 66 days after the luteinizing hormone (LH) surge or the first rise in serum progesterone to 2–3 ng/mL [7]. In queens, progesterone levels increase during the first three weeks after mating, reaching a peak between days 13 and 21 [8]. From approximately week 5 onward, concentrations begin to decline but do not return to basal levels until after parturition [9]. For this reason, progesterone monitoring is considered unreliable for estimating the timing of parturition in cats. Although progesterone is essential for pregnancy maintenance, the feline placenta—unlike that of the bitch—exhibits steroidogenic activity that contributes to sustaining progesterone levels throughout gestation [9]. Despite these endocrine differences, the stages of parturition in queens are comparable to those in bitches. Stage I typically lasts around two hours and involves uterine contractions and cervical dilation. Stage II is characterized by stronger uterine contractions and fetal delivery, and it may last up to six hours depending on litter size. Stage III involves placental expulsion, which may occur alternately with fetal delivery or after all fetuses are delivered [9].

While considerable attention has been devoted to the management of dystocia and delivery techniques, postnatal factors have gained increasing recognition for their influence on neonatal health and survival. These include environmental conditions, maternal behavior, and, importantly, the establishment of the neonatal gut microbiota [10]. The neonatal microbiota has emerged as a key contributor to early immune development, nutrient assimilation, gastrointestinal function, and long-term health outcomes [11]. Therefore, understanding how early microbial colonization occurs—and how it may be disrupted—has important implications for neonatal management and disease prevention in veterinary medicine.

## 2. Review Methodology

This review aimed to compile and critically analyze the scientific literature on the origin, colonization, and early development of the intestinal microbiota in neonatal companion animals, specifically domestic dogs and cats, with a primary focus on puppies and kittens. A comprehensive literature search was conducted between September 2024 and January 2026 using PubMed, Scopus, Google Scholar, and FARO (the electronic platform of the University of Las Palmas de Gran Canaria). Search terms included combinations of “puppy”, “kitten”, “microbiota”, “dysbiosis”, “lactation”, “neonates”, and “diarrhoea”.

Selected publications included peer-reviewed original research articles, review articles, and relevant book chapters published in English or Spanish within the last 20 years. Two older references (1998 and 1999) were retained due to their significant contributions to neonatal immunity and microbial colonization models. Inclusion criteria comprised studies addressing maternal microbiota (intestinal, vaginal, oral, or mammary), neonatal microbial colonization, and the impact of perinatal and environmental factors on neonatal health. Exclusion criteria included articles published before 1998 (unless justified) and non-peer-reviewed or scientifically unreliable sources. Reference lists of key papers were also screened to identify additional sources. All publications included in the final synthesis were analyzed and grouped thematically to facilitate discussion of (i) mechanisms of microbial transfer; (ii) the influence of delivery mode, lactation, maternal interaction, and environmental conditions; and (iii) the clinical implications of dysbiosis in neonates. As this study was based exclusively on previously published material, no ethical approval was required.

During manuscript preparation, generative artificial intelligence (GenAI) tools were used to assist in the drafting of original schematic figures based on an author-developed conceptual model and the information synthesized in this review. The GenAI outputs were used only as an initial layout/visual draft; all figure elements (terminology, structure, and scientific content) were subsequently reviewed, verified, edited, and finalized by the authors. No GenAI tools were used to generate or analyze primary data for this study.

## 3. Background and Context

**Figure 1:**
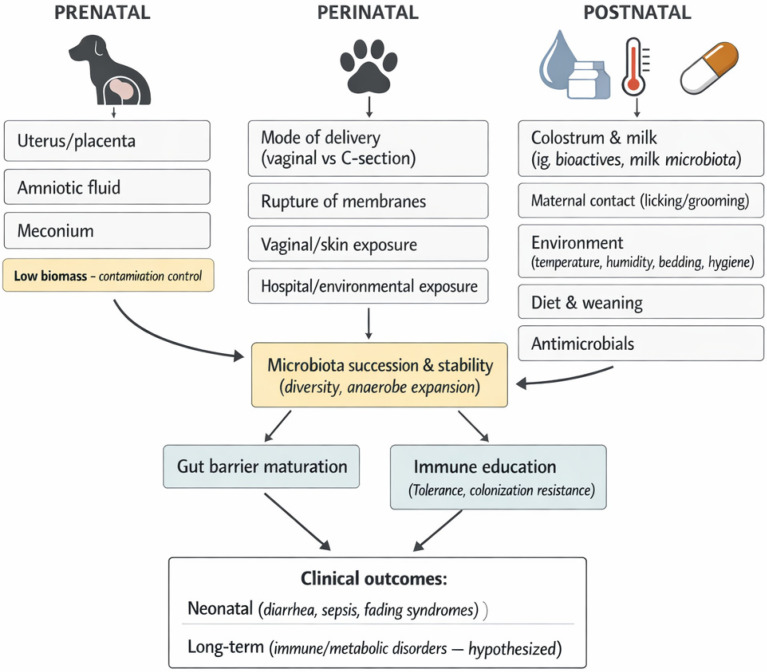
Determinants of neonatal gut microbiota establishment in puppies and kittens.

For over a century, it was widely accepted that the eutherian fetus develops in a sterile intrauterine environment, protected from microbial invasion by several mechanisms, including the anatomical separation of maternal and fetal circulations, the immune-privileged status of the placental trophoblast, and maternal immunotolerance during gestation [5]. However, recent research in both humans and other mammals has begun to question the long-held “sterile womb paradigm”, as bacterial communities have been detected in the uterus, placenta, amniotic fluid, and even in the meconium of healthy pregnancies [5]. Nevertheless, it is important to interpret these findings cautiously. Placental, amniotic-fluid, and meconium samples are typically low-biomass, and nucleic acid–based approaches are highly susceptible to reagent and environmental contamination, as well as batch effects, which may generate false-positive signals. Therefore, detection of microbial DNA does not necessarily imply the presence of viable, replicating microorganisms or true intrauterine colonization; robust conclusions require rigorous negative controls (e.g., sampling and extraction blanks), transparent contamination-filtering strategies, and, whenever feasible, complementary methods such as targeted quantitative polymerase chain reaction (qPCR), culture, microscopy, and/or viability-oriented assays. These discoveries support the idea that the establishment of the gut microbiome might start before birth and continue to develop afterwards. Advances in molecular methods have reinforced this perspective by enabling the detection of bacteria in reproductive tissues and fluids of various mammals, suggesting that maternal-to-fetal microbial transfer could occur during gestation [11]. However, species-specific differences in placentation and gestational physiology may limit direct extrapolation to companion animals [12] (Figure 1).

It is important to note, however, that some these findings are based on samples collected several days after birth, not immediately postpartum [5]. Puppies delivered by cesarean section may exhibit reduced initial microbial diversity and increased colonization by environmental opportunistic pathogens such as *Enterococcus*, *Enterobacter*, and *Klebsiella* spp., likely due to disruption of the vertical transmission of beneficial maternal bacteria such as *Bacteroides* and *Bifidobacterium* [5]. This suggests that incidental exposure to skin-associated and hospital-environment bacteria may significantly contribute to the early microbiota of cesarean-born neonates [13]. Comparable early-life microbiota datasets in cesarean-born kittens are currently limited, and feline-specific conclusions should therefore be considered preliminary.

Moreover, differences in sterility during parturition and the hygiene conditions between living between humans and dogs are additional key factors influencing the microbial colonization process [14]. In humans, early gut colonization is influenced by delivery mode and feeding practices. In dogs, available evidence suggests partially comparable early colonization dynamics; however, differences in physiology, husbandry, and environmental exposure limit direct extrapolation, and species-specific data—particularly in kittens—remain scarce [15].

Newborns are highly dependent on maternal care and have limited mobility, making maternal interaction a primary source of microbial exposure [16]. During nursing and grooming, the dam transfers components of her own microbiota to the pups [17]. Although puppies are raised in a relatively controlled environment, such as a whelping box or nest, they are also exposed to environmental microorganisms, including those introduced through regular human handling [16]. As in humans, diet plays a crucial role in shaping the gut microbiota of canine neonates [18]. Canine milk not only provides essential nutrients and immunoglobulins but also contributes to the establishment and modulation of the gut microbial population, particularly through the transmission of beneficial bacteria such as *Lactobacillus* spp. [15].

During the first weeks of life, the gut microbiota of puppies undergoes rapid changes. Initially dominated by *Firmicutes* (particularly *Clostridium*) these decrease over time [19]. In contrast, beneficial bacteria such as *Lactobacillaceae*, especially *Lactobacillus johnsonii*, increase, supported by maternal milk, which aids oligosaccharide digestion and modulates the intestinal environment. Early oxygen availability in the gut favors aerotolerant bacteria like *Proteobacteria* and *Bacteroidetes*. The latter, initially scarce, become predominant by the third week. This microbial succession supports gut maturation, immune regulation, and nutrient absorption, preparing the puppy for postnatal life [11].

Microbiota development is influenced by environmental factors, maternal health, and, critically, colostrum intake [19]. In dogs, only 5–10% of maternal antibodies cross the placenta and up to 25% in cats [20], leaving neonates nearly agammaglobulinemic. Therefore, early colostrum ingestion is vital, as immunoglobulin G (IgG) absorption through the intestinal epithelium is limited to the first 12–16 h. Although not fully understood, the enteromammary hypothesis suggests that colostral microbiota originates from the maternal gut [21]. Failure to ingest colostrum increases the risk of necrotizing enterocolitis and sepsis [22].

Recent evidence also suggests that microbial colonization in puppies may begin before birth, with bacteria identified in meconium (the first neonatal stool) [23]. Species such as *Staphylococcus*, *Streptococcus*, and *Neisseria zoodegmatis*, commonly found in the placenta and meconium, are also present in maternal oral and vaginal microbiota [13]. While early studies used matrix-assisted laser desorption/ionization time-of-flight mass spectrometry (MALDI-TOF MS), which only detects cultivable bacteria, metagenomic techniques now allow comprehensive profiling of both viable and non-viable microbes through microbial DNA analysis [14]. Against this background, the following section summarizes key concepts and competing hypotheses regarding early microbial acquisition in puppies and kittens, with emphasis on the strength and limitations of current evidence.

## 4. Neonatal Microbiota

### 4.1. Neonatal Microbiota: Concepts and Definitions

The microbiota refers to the community of microorganisms, including bacteria, archaea, fungi, viruses, protozoa, and other eukaryotes, that inhabit a specific environment within a host organism [24]. In contrast, the microbiome encompasses not only the microbial community itself, but also its collective genetic material and the interactions among microbes and with the host environment [24]. At birth, neonates possess an immature immune system that depends on early microbial exposure to trigger appropriate immunological development [25,26]. In this context, the study of microbiota in neonatal companion animals has gained increasing relevance, as it plays a critical role in shaping immune maturation, gastrointestinal function, and host–microbe symbiosis [27]. Understanding the progression and modulation of early-life microbial colonization is essential for improving neonatal health and survival in veterinary practice.

### 4.2. Theories on Intrauterine Microbial Exposure

Current knowledge regarding the development of the neonatal fecal microbiota in dogs and cats remains limited, although recent studies have begun to shed light on this early colonization process. The initial microbial community in neonatal carnivores is likely established through vertical transmission from the dam, either during parturition or via intrauterine exposure [28]. Following birth, the neonatal microbiota undergoes dynamic changes throughout the first month of life, influenced by maternal contact, feeding, and environmental exposure. The intestinal microbiota is understood as a complex community of commensal microorganisms that coexist with the host and contribute to immune education, digestive development, and mucosal protection [25].

Within this context, two competing theories have emerged regarding the origin of the initial microbial colonizers in neonates: the “sterile womb paradigm” and the “womb colonization hypothesis” [27]. The sterile womb paradigm refers to the belief that the uterine environment is free of microorganisms, and that colonization begins only at the time of birth, when the neonate is exposed to maternal skin, vaginal, and environmental microbiota [14,21]. This perspective also suggested that the presence of bacteria in colostrum or meconium was due to postnatal contamination, rather than in utero transmission. As such, for many years, meconium was assumed to be sterile, and maternal milk was not considered a source of endogenous microbial transfer.

In contrast, accumulating evidence over the past decade has challenged this traditional paradigm. Studies in both humans and animals have demonstrated the presence of bacterial DNA, and in some cases viable bacteria in the placenta, amniotic fluid, umbilical cord blood, and even in meconium collected immediately after birth, under sterile conditions. These findings support the womb colonization hypothesis, which proposes that maternal microbiota may translocate to the fetus via hematogenous routes, potentially through dendritic cells or translocation across the intestinal barrier to the mammary gland and placenta [14,25]. While the precise mechanisms remain under investigation, this hypothesis has prompted a reevaluation of the maternal-fetal microbiome axis and its implications for neonatal health. Nevertheless, these findings remain controversial, particularly in low-biomass samples, where contamination cannot be fully excluded. Importantly, most intrauterine/placental microbiome studies rely on nucleic acid–based methods in low-biomass samples, which are particularly susceptible to reagent and environmental contamination and batch effects. Therefore, detection of microbial DNA does not necessarily indicate the presence of viable, metabolically active microorganisms or true tissue colonization. Interpretation should be supported by rigorous negative controls (e.g., sampling blanks and extraction controls), transparent contamination-filtering strategies, and, whenever possible, complementary approaches such as targeted qPCR, culture-based methods, microscopy, and/or viability-oriented assays. These methodological constraints are especially relevant in companion animals, where available datasets in puppies are still limited and evidence in pre-weaning kittens is scarce.

Understanding the timing and mechanisms of microbial colonization is highly relevant in the field of veterinary neonatology. The neonatal intestinal microbiota has emerged as a critical determinant of early-life health, playing a pivotal role in immune system maturation, resistance to pathogenic colonization, and susceptibility to both gastrointestinal and systemic diseases [29]. Given the high morbidity and mortality rates observed in neonates (particularly in the first weeks of life), there is a growing need to elucidate the factors that shape microbial development in puppies and kittens. These include delivery mode, colostrum ingestion, maternal microbiota composition, and environmental hygiene.

### 4.3. Relevance for Veterinary Neonatology

The increasing interest in neonatal microbiome research underscores its potential to inform preventive strategies and therapeutic interventions. From optimizing maternal health and perinatal care to developing targeted probiotic therapies, a deeper understanding of the neonatal microbiota could significantly improve survival rates and long-term health outcomes in companion animals [30]. In this context, the present review seeks to synthesize current scientific evidence regarding the origin, modulation, and clinical relevance of the neonatal microbiota, with the aim of guiding future research and improving clinical practices in veterinary neonatology.

### 4.4. Microbial Colonization in Early Life

The establishment of microbial communities during early life is a critical developmental process, especially within the gastrointestinal tract, which hosts most the body’s commensal microbes. This early colonization plays a fundamental role in shaping immune and metabolic pathways, supporting neonatal immune development, providing protection against enteric pathogens, and influencing long-term health outcomes [31,32].

Historically, microbial colonization was believed to begin only at birth, as per the sterile womb paradigm [33]. However, this view has been increasingly challenged by recent findings in both humans and animals, including canines, which suggest that microbial acquisition may begin in utero. The identification of bacterial DNA, RNA, and even viable organisms in placenta, amniotic fluid, the fetal gastrointestinal tract, and meconium of neonates has led to a paradigm shift [34]. The placenta, once considered sterile, has been proposed to harbor microbial DNA in several species, and the presence of microbes in meconium further supports the intrauterine origin of at least part of the neonatal microbiota [23,28,35]. Notably, available longitudinal data are substantially more abundant in puppies than in kittens, particularly during the pre-weaning period. Accordingly, in the following sections we explicitly differentiate feline-specific evidence from hypotheses or extrapolations based on canine and human studies.

In puppies, the fecal microbiota undergoes rapid and dynamic changes during the first two months of life. Between days 2 and 56, significant phylogenetic shifts occur across all taxonomic levels. Initially, *Firmicutes* dominate the microbiota, but by day 21, *Bacteroidetes* and *Fusobacteria* emerge alongside *Firmicutes* as co-dominant groups [19]. These transitions are accompanied by increases in microbial diversity and richness and reflect the progressive maturation of the gastrointestinal tract and its functional capacity to resist environmental pathogens. By day 42, microbial communities begin to stabilize, indicating the establishment of a more resilient and balanced intestinal ecosystem [28].

In kittens, early-life gut microbiota development has been less extensively characterized, and most available datasets focus on the weaning transition rather than the immediate neonatal period [36]. The limited evidence available indicates that the kitten gut microbiota also undergoes marked compositional changes around weaning, but neonatal trajectories and drivers (e.g., delivery mode, colostrum intake, and maternal contact) remain insufficiently defined and require dedicated longitudinal studies.

#### 4.4.1. Maternal Microbiome During Gestation and Intestinal Microbiota

The mother represents the primary source of microbial inoculum for the neonate. During gestation, the maternal microbiota undergoes physiological changes that support fetal development and may influence microbial transmission [22]. Although specific data on pregnant bitches remain limited, studies indicate that the dominant phyla in the fecal microbiota of healthy adult dogs include *Fusobacterium*, *Bacteroidetes*, and *Firmicutes* [29]. Toward late gestation, the maternal gut microbiota may shift, with reports describing increased relative abundance of *Firmicutes* and *Proteobacteria* and changes in lactic-acid bacteria in some settings. Similar broad trends, particularly an increase in *Proteobacteria* and *Actinobacteria* and reduced within-sample diversity, have been described in human pregnancy, although the magnitude and direction of changes vary across cohorts and methodologies [28,37,38].

#### 4.4.2. Vaginal Microbiota

The vaginal microbiota of pregnant bitches may also contribute to vertical transmission of pathogens. It is known to fluctuate throughout the estrous cycle and gestation, with genera such as Neisseria, Haemophilus, and Enterococcus tending to predominate toward the end of pregnancy [28]. The endometrial microbiota is composed mainly of *Proteobacteria*, *Firmicutes*, *Actinobacteria*, and *Bacteroidota*, while species commonly isolated from the vaginal mucosa include *Lactobacillus*, *Staphylococcus*, *Mycoplasma*, and *Streptococcus* [28]. These bacteria may be involved in the initial colonization of the neonate during vaginal birth.

Literature concerning the vaginal microbiota in felines remains limited. However, available studies confirm that the vaginal microbial community of healthy queens is primarily composed of bacteria belonging to the phyla Proteobacteria, Firmicutes, Bacteroidota, and Actinobacteria [39]. At the genus level, commonly identified taxa include Escherichia-Shigella, Streptococcus, Pasteurella, Bacteroides, and Staphylococcus. This bacterial profile appears to differ from that reported for healthy bitches in the available literature; however, it should be noted that these canine and feline data come from different studies using distinct populations and methodologies. Therefore, cross-species comparisons should be interpreted cautiously, and direct, standardized head-to-head studies are needed to confirm true interspecies differences [39]. These interspecies differences may reflect variations in reproductive physiology, vaginal pH, hormonal cycling, and environmental exposure, and they highlight the importance of species-specific research in understanding microbial contributions to reproductive health.

#### 4.4.3. Oral and Skin Microbiota

The oral microbiota of the dam is another potential source of microbial transmission, especially given the frequent licking and grooming of neonates [15]. The canine oral cavity harbors a highly diverse microbial community, with over 600 identified species. In pregnant bitches, dominant taxa include *Neisseria zoodegmatis*, *Actinomyces canis*, and *Staphylococcus* spp. [13]. The notable similarity between some bacterial populations in the placenta and the maternal oral cavity raises the possibility of hematogenous translocation, a mechanism already proposed in human medicine [5]. Each dam possesses a unique microbial fingerprint that significantly influences the structure and composition of her offspring’s microbiota [15]. This maternal individuality highlights the importance of maternal health and microbial status during gestation and lactation. Modulating the maternal microbiota through diet, prebiotics, or probiotics during pregnancy may offer a promising strategy to enhance neonatal microbial colonization and promote optimal immune and gastrointestinal development. Despite this, the clinical implications of early-life mycobiome alterations in puppies and kittens remain poorly characterized compared with bacterial dysbiosis, and are therefore discussed only briefly.

**Figure 2:**
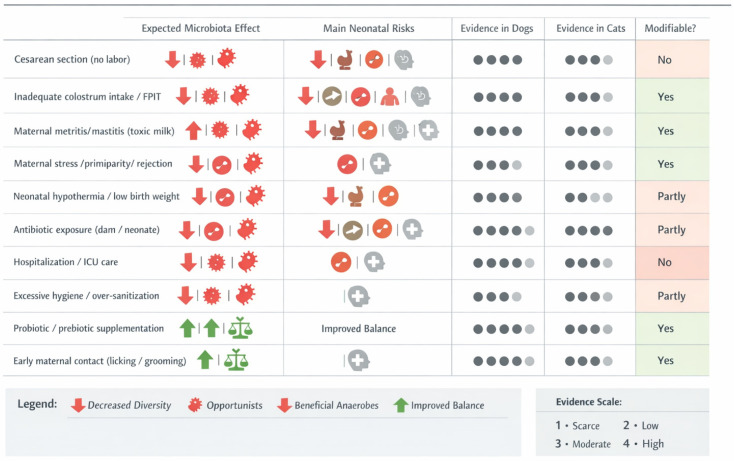
Perinatal and early-life factors influencing neonatal guy microbiota in puppies and kittens. The number of dark gray circles determines the level of evidence (Evidence scale) for each species. 1 represents low evidence and 4 the highest level.

In felines, both the oral and cutaneous microbiota show notable individual variability in their composition. On the skin, the predominant bacterial phyla include Proteobacteria, Firmicutes, Actinobacteria, and Bacteroidetes, although their relative abundance may differ between individuals [40]. Additionally, the skin of both cats and dogs is colonized by a fungal microbiota, with Dothideomycetes being the most frequently reported fungal class [40]. The oral microbiota in cats is highly diverse and has been extensively studied, particularly in the context of infectious diseases affecting the oral cavity. Notably, investigations have revealed the presence of fungal colonies in the mouth that appear to be associated with the species found on the skin, suggesting possible microbial overlap between these anatomical regions [40]. While the oral microbiota shares similarities with that of the skin, it is characterized by a higher relative abundance of Bacteroidetes, which appear to predominate in the feline oral environment. Regardless of the relative contribution of prenatal routes, the immediate postnatal period represents a major window for microbial transfer, particularly through lactation and maternal contact, as discussed below (Figure 2).

## 5. Theories of Maternal to Fetal Microbial Transfer

The precise mechanisms by which maternal microorganisms reach the fetal environment remain incompletely understood. Several pathways have been proposed, including ascending migration of bacteria from the vaginal canal to the placenta, hematogenous dissemination from the maternal oral cavity, and translocation from the maternal gut via immune cells such as dendritic cells [28]. Among these, vertical transmission through the vaginal canal during parturition appears to be the most robustly supported route. However, the translocation of bacteria into the bloodstream is known to increase during pregnancy, potentially facilitating low-level bacteremia and subsequent microbial colonization of the placenta [28,41,42,43].

The detection of bacterial DNA and, in some cases, viable microorganisms in healthy placental tissue has raised important questions regarding the potential physiological roles of the placental microbiota [44]. It has been hypothesized that controlled exposure to microbial antigens in utero may contribute to early immune education and tolerance, shaping the neonate’s immunological landscape prior to birth. Although the viability of many detected bacteria remains uncertain due to reliance on nucleic acid-based methods, even non-viable bacterial components may exert immunomodulatory effects through interaction with pattern recognition receptors [23].

In the feline species, bacterial genetic material has also been identified within the uterus of healthy pregnant queens. Among the microorganisms isolated, *Burkholderia cepacia* has been reported in feline placental samples [42]. However, given the low biomass of these samples, such findings should be interpreted cautiously and confirmed with stringent contamination controls and viability-oriented methods before inferring true colonization. This Gram-negative bacterium is of particular concern due to its well-documented multidrug resistance and its ability to survive and proliferate in antiseptic environments [42]. These findings further emphasize the need for deeper investigation into the nature, origin, and role of the uterine microbiota in felines.

## 6. Lactation and Maternal Interaction

Following birth, lactation and maternal interaction constitute a critical window during which neonatal microbial colonization continues and accelerates [11,14]. This period is characterized by intensive maternal investment and high energy demands, during which the dam or queen provides not only essential nutrients but also immunological and microbial stimuli crucial for early life development [45]. Maternal milk is a key vector for microbial transmission, both through direct contact during suckling and via microor-ganisms present in the milk itself. The so-called entero-mammary pathway suggests that maternal gut microbes may translocate to the mammary gland via immune cell traf-ficking, particularly through mesenteric lymph nodes, and be delivered to the neonate via colostrum and milk [14].

Interestingly, the microbial composition of neonatal meconium and maternal co-lostrum have been found to be similar, supporting the hypothesis that mothers may “pre-program” the initial microbial communities of their offspring [14,22]. This maternal imprinting likely contributes to the observation that microbial diversity is lower within litters compared to between litters, emphasizing the dam’s influence on the establishment of early microbial profiles.

However, maternal factors such as primiparity, psychological stress, or cesarean section without preceding labor can negatively influence maternal behavior, potentially resulting in rejection or inadequate care of the neonates [22]. Such disruptions can lead to insufficient colostrum intake, resulting in the neonatal triad (hypothermia, hypoglyce-mia, and dehydration) and significantly increasing the risk of neonatal mortality [10]. For this reason, daily monitoring of neonatal weight gain is considered a sensitive and non-invasive indicator of adequate milk intake and general neonatal well-being [45].

## 7. Environmental Factors

Environmental conditions play a decisive role in the health, microbial colonization, and survival of neonates, as well as in the well-being and maternal behavior of the dam. Suboptimal management of environmental parameters has been directly associated with increased neonatal morbidity and mortality [10]. The maternity area should be quiet, free of excessive noise, foot traffic, and drafts, and the mother should be gradually introduced to it at least two weeks prior to parturition. This early familiarization reduces stress-related behaviors that can interfere with maternal care, milk production, and neonatal acceptance [10].

Temperature and humidity must be carefully controlled, as neonates possess limited thermoregulatory capacity during the first weeks of life. Recommended ambient temperatures range between 29–32 °C during the first week of life in the absence of the dam, and 20–24 °C if the dam is present. Relative humidity should be maintained between 50–60% to prevent dehydration and respiratory compromise [10]. Deviations from these parameters can result in either hypothermia or hyperthermia, both of which are associated with increased mortality [10].

Hygiene is another essential consideration during the neonatal period. While rigorous sanitation protocols reduce the risk of infectious disease, excessive or indiscriminate cleaning practices may inadvertently disrupt beneficial microbial exposure, potentially altering the development of the neonate’s immune system and increasing susceptibility to allergic or autoimmune conditions later in life [14]. In addition, bledding should be clean, dry, absorbent, and thermally insulating. Inadequate bedding can lead to conductive heat loss, contamination, and increased risk of infection.

In addition to physical and environmental parameters, maternal behaviors such as licking play a fundamental role in shaping the early microbiota of neonates [14]. In vaginal delivery, colonization begins as early as the rupture of the amniotic membrane. Under normal circumstances, the membrane remains intact until birth, at which point the dam instinctively tears it open, severs the umbilical cord, and vigorously licks the neonate [25]. This process is not only essential for stimulating respiration and circulation, but also exposes the neonate to the maternal microbiota via the skin, oral cavity, and vaginal secretions [14,16]. The act of licking, combined with the high humidity and warmth of the whelping environment and the close physical proximity among littermates, creates an occlusive microenvironment that promotes microbial proliferation and colonization of the skin, oronasal cavity, and gastrointestinal tract [15]. These early postnatal interactions represent a vital extension of maternal microbial transfer and contribute to the establishment of a stable and resilient neonatal microbiome. Because early colonization is sensitive to these management factors, disruption of normal microbial succession may contribute to neonatal disease susceptibility, as outlined in the next section.

## 8. Association with Neonatal Diseases

### 8.1. Fading Puppy/Kitten Syndrome (FPS)

Fading Puppy Syndrome (FPS) is a fatal neonatal condition characterized by the progressive decline and eventual death of seemingly healthy puppies within the first three weeks of life [46]. It is estimated to affect up to 30% of litters, with mortality rates approaching 100% in affected individuals. Most deaths occur within the first seven days postpartum [22,47]. The etiology of FPS is multifactorial and remains incompletely understood, but it is believed to involve a combination of infectious, environmental, immunological, and metabolic factors [48]. While alterations in intestinal microbiota have not been extensively studied in the context of FPS, emerging evidence suggests that intestinal dysbiosis is associated with FPS and may be involved in its pathophysiology [47]. Importantly, given the observational design, these microbial patterns should be interpreted as concomitant findings and do not establish causality.

Puppies diagnosed with FPS exhibit a distinct pattern of rectal microbial diversity, notably a higher Proteobacteria-to-Firmicutes ratio, increased abundance of Pasteurellaceae, and reduced populations of *Clostridia* and *Enterococcus* spp. [47]. This microbial shift may result in a less resilient intestinal ecosystem, favoring the proliferation of opportunistic pathogens and impairing mucosal barrier function. Such dysbiosis could plausibly facilitate bacterial translocation across the immature intestinal epithelium, triggering systemic infection or a dysregulated immune response [22]. Additionally, the maternal contribution to microbial colonization must be considered. Inadequate microbial composition in the dam’s colostrum, potentially due to disease, stress, or nutritional deficiencies, may impair the initial microbial seeding of the neonate and predispose to FPS [21]. However, further studies are needed to clarify the specific role of microbial factors in its pathophysiology and to identify potential prophylactic interventions.

**Figure 3:**
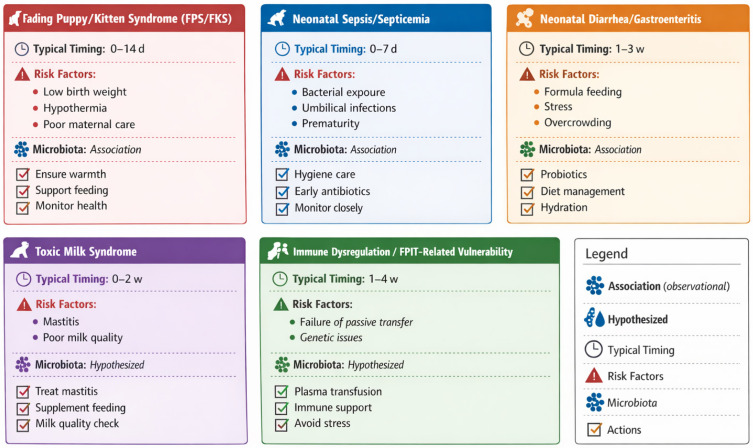
Neonatal conditions associated with early life dysbiosis.

A similar syndrome to fading puppy syndrome has been described in kittens and is characterized by the progressive deterioration of one or more neonates within a litter that initially appears healthy. Affected kittens gradually become lethargic, weaken, and eventually die, typically within the first two weeks of life [49]. The etiology is often multifactorial and may include infectious, toxic, traumatic, metabolic, or genetic causes. In most cases, diagnosis is challenging due to the nonspecific nature of clinical signs. Among non-infectious causes, neonatal isoerythrolysis is considered one of the most frequent. This condition results from an incompatibility between the blood type of the queen and that of her offspring, leading to hemolysis after the neonate ingests colostrum containing maternal anti-A antibodies. It is more commonly observed in certain breeds predisposed to blood type B, such as British Shorthair or Devon Rex [50]. Since the maternal antibodies target the neonate’s red blood cells, clinical signs may include jaundice, hemoglobinuria, weakness, and sudden death. Early prevention through blood typing of breeding animals is critical in at-risk matings (Figure 3).

### 8.2. Sepsis and Septicemia

Sepsis is the leading cause of neonatal mortality during the first three weeks of life in canines [10]. It is defined as a systemic inflammatory response syndrome (SIRS) triggered by an acute and generalized bacterial infection, which may progress to septic shock characterized by circulatory failure, persistent hypotension, and multiple organ dysfunction syndrome (MODS). By contrast, bacteremia refers to the presence of bacteria in the bloodstream without necessarily inducing organ failure [10]. Clinically, neonatal sepsis is often insidious, with early signs including lethargy, weak or absent suckling reflex, failure to gain weight, and the presence of the so-called “neonatal triad”: hypothermia, hypoglycemia, and dehydration [51]. Diarrhea is frequently observed—reported in up to 93% of septic neonates in some studies—although it is not pathognomonic [10].

The dam is considered the primary source of infection in 87.6% of sepsis cases, with transmission occurring either in utero (68%), during birth, or postnatally. The uterine environment is the most common origin of pathogenic bacteria, followed by colostrum/milk and maternal oral secretions [10]. Across published reports on neonatal canine sepsis, Escherichia coli is consistently among the most frequently isolated bacteria, commonly recovered from the endometrium and/or mammary secretions of the dam. Other commonly reported isolates include β-hemolytic *Streptococcus* spp. and *Staphylococcus* spp. [10]. Successful passive transfer of immunity via colostrum remains the cornerstone of neonatal defense. In cases where colostrum intake is insufficient or absent, administration of hyperimmune plasma or serum may provide critical immunoglobulin support, improving survival outcomes [10].

In neonatal kittens, infectious diseases are the leading cause of sudden death during the first weeks of life [49]. Among the most frequently isolated bacteria in fatal cases are *Escherichia coli*, *Streptococcus* spp., *Pasteurella* spp., and *Staphylococcus* spp. Many of these microorganisms are part of the normal commensal flora of the gastrointestinal tract, skin, or mucous membranes [49]. However, in the presence of predisposing factors, such as hypothermia, inadequate colostrum intake, or maternal neglect, these bacteria can adopt a pathogenic role, leading to rapid systemic infection and sepsis [50]. The immature immune system of the neonate, coupled with environmental or physiological stress, increases susceptibility to opportunistic infections.

### 8.3. Neonatal Diarrhea and Gastroenteritis

Gastrointestinal disorders, particularly diarrhea, are among the most common clinical problems in neonates and young animals. In puppies, diarrhea has been reported in up to 60% of clinically ill individuals [52], and in kittens, it is the second leading cause of death in shelter populations [35]. The etiology of neonatal diarrhea is multifactorial. Immature immune responses, stress caused by weaning, abrupt dietary transitions, loss of maternally derived antibodies, and increased environmental exposure to pathogens all contribute to heightened susceptibility [35]. These factors disrupt the delicate balance of the developing intestinal microbiota, often leading to dysbiosis and inflammation.

In recent years, probiotic supplementation has emerged as a promising prophylactic and therapeutic strategy. Probiotics can promote colonization resistance, modulate local and systemic immune responses, enhance mucosal barrier function, and improve nutrient digestion and absorption [52]. A notable study done in puppies, demonstrated that maternal dietary supplementation with prebiotics and probiotics (including mannan-oligosaccharides (MOS), fructo-oligosaccharides (FOS), Enterococcus faecium, and Lactobacillus acidophilus), significantly reduced the incidence and severity of gastroenteritis in their offspring [52]. This effect is attributed to two mechanisms: (1) enhancement of colostrum quality through immunoglobulin enrichment via the entero-mammary pathway, and (2) vertical transfer of a more balanced and protective maternal microbiota. Enterococcus faecium has demonstrated in vitro inhibitory activity against various enteropathogens and stimulates intestinal IgA production, contributing to enhanced mucosal immunity in neonates [52] (Figure 3).

### 8.4. Toxic Milk Syndrome

Toxic Milk Syndrome is a non-infectious but clinically significant condition in neonates, resulting from the ingestion of maternal milk contaminated with bacterial toxins, most associated with subclinical or clinical mastitis and/or metritis in the dam [22]. Although the milk may appear macroscopically normal, it can contain endotoxins or inflammatory mediators that are absorbed through the immature gastrointestinal tract of the neonate, leading to systemic effects. Clinically, affected puppies may present with sudden weakness, vomiting, diarrhea, and failure to thrive within hours of ingesting contaminated milk. In severe cases, rapid deterioration and death can occur [10]. Diagnosis is often presumptive, based on clinical signs and the identification of maternal uterine or mammary pathology. Preventive strategies include close postpartum monitoring of the dam for signs of mastitis or metritis, proper hygiene in the whelping area, and early veterinary intervention when abnormalities are suspected [53] (Figure 3).

### 8.5. Immune Dysregulation

Passive immune transfer is essential for neonatal survival in species with limited transplacental immunoglobulin passage, such as dogs and cats [53]. The extent to which maternally derived antibodies reach the fetus is determined by the structural type of the placenta. In carnivores, the endotheliochorial placenta allows only minimal transplacental immunoglobulin transfer—estimated at 5–10% in dogs and up to 25% in cats—leaving neonates largely dependent on the ingestion of colostrum for effective immune protection [54,55]. This dependence is compounded by the immaturity of the neonatal immune system, which lacks both cellular and humoral competence during the early postnatal period. As such, timely colostrum intake is critical to ensure adequate immunological defense, particularly against opportunistic and environmental pathogens [22].

Canine colostrum is rich in immunoglobulins, predominantly IgA, but also IgG and IgM, as well as a wide array of bioactive compounds, including nutrients, lactoferrin, lysozyme, leukocytes, cytokines, hormones (e.g., cortisol, insulin, thyroxine, growth hormone), microbiota, and growth factors such as epidermal growth factor (EGF), insulin-like growth factors (IGF), and nerve growth factor (NGF) [22,56]. Many of these components not only contribute to immune development but also play vital roles in gut maturation, neurodevelopment, and metabolic regulation. The immunoglobulins present in colostrum and milk are produced by plasma cells residing in the mammary gland. In murine models, these plasma cells originate from gut-associated lymphoid tissue (GALT), particularly Peyer’s patches, and migrate to the mammary gland via the bloodstream—supporting the concept of the entero-mammary pathway [57].

The window for antibody absorption in neonates is narrow and highly time dependent. Puppies and kittens can absorb immunoglobulins via specialized enterocytes only during the first 12–16 h of life, with peak permeability occurring within the first 8–12 h [10,58]. After this period, intestinal epithelial cells undergo closure, characterized by tight junction formation, effectively terminating systemic immunoglobulin absorption [10,22]. Failure of passive immune transfer (FPIT) has been reported in approximately 17.4% of canine neonates. Serum IgG concentrations below 2.3 g/L are considered predictive of increased mortality; nearly 40% of puppies with IgG levels under this threshold die within the first 21 days after birth [10,22]. FPIT is strongly associated with increased susceptibility to life-threatening conditions such as septicemia and necrotizing enteric disease. Multiple factors influence the success of passive immunity transfer, including:

- The volume and immunological quality of colostrum, which depend on maternal health, nutrition, and vaccination status [59].
- Maternal behavior and lactation ability (e.g., agalactia, hypogalactia, rejection) [59].
- Neonatal viability (e.g., birth weight, strength of suckling reflex, thermoregulation) [10].
- Neonatal hypothermia and low birth weight can impair the suckling reflex, leading to insufficient colostrum intake. Similarly, maternal malnutrition or lack of prepartum immunization may compromise colostrum quality. These variables underscore the importance of comprehensive peripartum management [10].

Emerging evidence supports the role of maternal nutrition, particularly dietary supplementation with prebiotics and probiotics during gestation, in enhancing colostrum quality and immunological potential [60,61]. This is mediated through modulation of the maternal gut microbiota and subsequent microbial and immunological enrichment of the colostrum and milk, via the entero-mammary link [52,56]. This maternal-neonatal microbial continuum may serve as a modifiable factor to improve neonatal immune competence and reduce early-life morbidity and mortality.

### 8.6. Other Associated Conditions

Beyond acute infectious diseases, early-life alterations in the gut microbiota have been increasingly linked to the development of chronic, immune-mediated, metabolic, and neurodevelopmental disorders in both veterinary and human medicine [18,62,63]. In companion animals, primarily in dogs, conditions such as obesity, allergic diseases, atopic dermatitis, asthma-like syndromes, metabolic imbalances, and even neurodegenerative processes have been associated with dysbiosis during the neonatal period [14,28]. Specifically in dogs, atopic dermatitis—a chronic inflammatory skin condition frequently encountered in clinical dermatology—has been associated with alterations in gut microbial composition [64]. Affected animals often exhibit reduced diversity and stability of intestinal microbiota, which may compromise mucosal immune tolerance and promote systemic inflammatory responses [14]. This suggests a gut–skin axis in canine health, like that described in humans.

In human neonates, the disruption of natural microbial transmission during birth has been extensively studied. Infants delivered via Cesarean section miss the exposure to maternal vaginal and fecal microbiota, which significantly alters the initial colonization process [65]. These infants exhibit increased colonization by opportunistic bacteria such as *Enterococcus*, *Enterobacter*, and *Klebsiella*—often derived from the hospital environment—and reduced abundance of beneficial genera such as *Lactobacillus* and *Bifidobacterium* [21]. In humans, Cesarean delivery has been associated with an altered early colonization pattern and with a higher incidence of some chronic immune-mediated conditions in epidemiological studies (e.g., asthma and celiac disease) [64,66]. However, causality and mechanisms remain debated. Comparable long-term cohort studies are currently lacking in dogs and cats; therefore, any similar associations in veterinary species should be considered hypothetical and warrant dedicated longitudinal research. The cumulative evidence from both veterinary and human research underscores the far-reaching importance of early microbial colonization. It serves not only as a defense mechanism against pathogens in the neonatal period, but also as a regulatory factor in immune, metabolic, and even neurological development.

### 8.7. Fungal Communities (Mycobiome) and Clinical Relevance (Proposed)

Although this review primarily addresses bacterial gut microbiota, fungal communities (the mycobiome) are also part of early-life microbial exposure in puppies and kittens. In dogs and cats, fungi are well-recognized contributors to skin and mucosal disease (e.g., Malassezia-associated dermatitis/otitis and opportunistic Candida overgrowth), and early-life immune immaturity may increase susceptibility when normal microbial community structure is disrupted. However, feline and canine neonatal data linking intestinal fungal dysbiosis to clinical outcomes (e.g., diarrhea, sepsis, or poor growth) remain scarce, partly due to limited routine profiling of fungi in low-biomass neonatal samples and the strong influence of environment, diet, and antimicrobial exposure. Antibiotic administration, hospitalization, and intensive hygiene measures may reduce bacterial competitors and theoretically favor fungal overgrowth, but this hypothesis requires dedicated studies in neonates. Future work should integrate bacterial and fungal profiling (e.g., 16S + ITS sequencing) to clarify whether specific fungal signatures are associated with high-risk neonatal scenarios and to define clinically relevant intervention targets.

## 9. Knowledge Gaps and Future Directions

Despite growing interest, major gaps remain in companion animal neonatal microbiome research. First, evidence in kittens, especially during the pre-weaning period, is limited, and most available data cannot be directly extrapolated from canine or human studies. Second, several studies rely on small sample sizes and heterogeneous designs (different sampling sites, timepoints, diets, and housing), which hinders comparability and limits causal inference. Third, studies addressing potential prenatal microbial exposure are particularly vulnerable to contamination bias due to the low biomass of reproductive tissues and meconium; standardized protocols and rigorous negative controls are essential. Finally, the clinical relevance of early dysbiosis in puppies and kittens (e.g., links to sepsis, diarrhea, fading syndromes, or later immune-mediated diseases) requires well-designed longitudinal cohorts integrating microbiome profiling with clinically meaningful outcomes. Future research should prioritize species-specific, longitudinal studies from birth through weaning, incorporate metadata on perinatal management (delivery mode, colostrum intake, antimicrobial exposure, hygiene), and evaluate targeted interventions (maternal diet modulation, pre/probiotics) using randomized controlled designs where feasible.

## 10. Conclusions

The establishment and development of the gut microbiota during the neonatal period is a fundamental process that influences immune maturity, gastrointestinal function, and long-term health in puppies and kittens. This review highlights the maternal, perinatal, and environmental factors that shape microbial colonization, including delivery mode, colostrum intake, maternal health, and hygiene conditions. Disruptions in early microbial transmission, such as cesarean delivery or inadequate maternal care, have been associated with increased risk of neonatal diseases, including sepsis, diarrhea, and immune dysregulation. Interventions such as improving maternal nutrition, promoting colostrum intake, and considering probiotic supplementation may serve as promising strategies to support healthy microbial colonization and reduce neonatal morbidity and mortality. Future studies in puppies and especially in pre-weaning kittens should prioritize standardized sampling/reporting and strict contamination control strategies in low-biomass specimens to clarify the timing of initial colonization and its clinical relevance. Future studies should prioritize longitudinal, species-specific cohorts in kittens sampled immediately after birth (e.g., within the first 0–6 h, 24 h, 48 h, and 72 h) and followed through weaning, to define early succession patterns and the relative contribution of delivery mode, colostrum intake, and maternal contact.

In parallel, randomized controlled trials should evaluate targeted interventions in high-risk scenarios—such as cesarean-delivered puppies—including defined probiotic/prebiotic strains and dosing regimens, using clinically meaningful endpoints (e.g., diarrhea incidence, sepsis, growth rate, and survival) alongside microbiome and immune readouts.

## Data Availability

No external datasets or repositories are needed to review the findings.
